# Anatomy based corridors to the infratemporal fossa: Implications for endoscopic approaches

**DOI:** 10.1002/hed.26055

**Published:** 2019-12-27

**Authors:** Lifeng Li, Nyall R. London, Daniel M. Prevedello, Ricardo L. Carrau

**Affiliations:** ^1^ Department of Otolaryngology‐Head & Neck Surgery, Beijing Tongren Hospital Capital Medical University Beijing China; ^2^ Department of Otolaryngology‐Head & Neck Surgery The James Cancer Hospital at the Wexner Medical Center of The Ohio State University Columbus Ohio; ^3^ Department of Otolaryngology‐Head & Neck Surgery Johns Hopkins School of Medicine Baltimore Maryland; ^4^ Division of Otolaryngology‐Head and Neck Surgery National Institute on Deafness and Other Communication Disorders NIH, Bethesda Maryland; ^5^ Department of Neurological Surgery The James Cancer Hospital at the Wexner Medical Center of The Ohio State University Columbus Ohio

**Keywords:** endoscopic Denker's approach, infratemporal fossa, muscles of mastication, pterygoid muscle, transpterygoid

## Abstract

**Background:**

The infratemporal fossa (ITF) represents an area densely packed with neurovascular structures within irregular boundaries. The goal of this study was to classify the ITF into zones corresponding to its anatomical spaces and the order in which they are encountered during an endonasal approach (anteroposterior axis).

**Methods:**

Six cadaveric specimens (12 sides) with injected colored latex were dissected. Following an endoscopic medial maxillectomy and Denker's approach, a progressive exploration of the masticator space and upper parapharyngeal space was completed. A classification of the ITF based on well‐defined spaces was ascertained.

**Results:**

The ITF was divided into five zones:

- Zone 1 (retromaxillary space)—space lying between the posterolateral wall of the maxillary sinus and the temporalis and pterygoid muscles.
- Zone 2 (superior interpterygoid space)—area including the superior head of the lateral pterygoid muscle, V_3_, and foramen ovale.
- Zone 3 (inferior interpterygoid space)—includes the inferior head of lateral pterygoid muscle, medial pterygoid, and temporalis muscles, and the space enclosed by these muscles.
- Zone 4 (temporo‐masseteric space)—space lateral to the temporalis muscle (comprising fat mostly).
- Zone 5 (tubopharyngeal space)—includes the Eustachian tube, tensor, and levator veli palatini muscles, and structures in upper parapharyngeal space.

**Conclusion:**

The ITF can be visualized as five zones based on spaces enclosed by the masticator muscles and upper parapharyngeal structures. This novel classification system is useful to guide endoscopic approaches to the ITF, while decreasing the potential for injury of neurovascular structures and pterygoid muscles.

## 1 INTRODUCTION

The infratemporal fossa (ITF) is an anatomic space with irregular boundaries, encompassing the masticator and upper parapharyngeal spaces (UPPS) and located below the floor of the middle cranial fossa.^1^ In turn, the masticator space includes the medial and lateral pterygoid muscles, the tendon of the temporalis muscle, internal maxillary artery, maxillary (V_2_) and mandibular (V_3_) branches of the trigeminal nerve, the tensor and levator veli palatini muscles, and the Eustachian tube.^2^ The styloid diaphragm, formed by the styloid aponeurosis, divides the UPPS into pre‐ and post‐styloid compartments.^3^

Lesions that involve the ITF include those that originate within the space, for example, schwannomas, and tumors that invade from adjacent structures, such as inverted papilloma, juvenile angiofibroma, adenoid cystic carcinoma, and squamous cell carcinoma.^4^, ^5^, ^6^, ^7^ Its complex neurovascular anatomy has engendered the development of multiple classical surgical approaches following a lateral to medial direction (ie, pre‐ and postauricular, transcervical, transparotid, or combined approaches), and anterior (ie, trans‐maxillary) or medial (ie, transoral) routes.^8^, ^9^, ^10^ Following the development of endoscopic techniques and instrumentation, endoscopic endonasal or transoral approaches for the removal of benign and select malignant lesions of the ITF, have gained popularity.^11^, ^12^, ^13^ Of note, the removal of tumors locating at pre‐styloid parapharyngeal space via an endoscopic transoral approach with the assistance of robotics has achieved satisfying outcomes.^14^

The endoscopic transpterygoid approach to the ITF has been well described and, in select cases, it can yield similar outcomes to open approaches with decreased morbidity.^1^, ^2^, ^15^ However, it may require the sacrifice of multiple neurovascular bundles within the pterygopalatine fossa.^1^ Moreover, adequate exposure of lesions infiltrating the Eustachian tube or UPPS require the mobilization or removal of the soft tissue contents of the pterygopalatine fossa.^16^, ^17^ This leaves significant undesirable sequelae, as sacrificing the vidian and greater palatine nerves in pterygopalatine fossa may cause post‐operative xerophthalmia and a variety of sensory dysfunctions of the palate (ie, hypoesthesia, anesthesia or deafferentation pain).^10^, ^18^ Furthermore, the lateral and medial pterygoid muscles also need to be transected or resected when approaching the lateral ITF (masticator space), which may contribute to postoperative trismus.^1^

Cadaveric dissections have helped to identify several potential spaces enclosed by the pterygoid and temporalis muscles.^19^ Therefore, we hypothesized that these may provide safe surgical corridors to and within the ITF. The goal of this study was to develop a sequential ITF dissection illustrating surgical corridors, which may be beneficial when planning endoscopic approaches to specific lesions; therefore, resulting in an efficient surgical exposure with maximal preservation of neurovascular structures and pterygoid muscles.

## 2 MATERIALS AND METHODS

An endoscopic medial maxillectomy was combined with an endoscopic Denker's approach to the ITF in six adult latex injected cadaveric specimens (12 sides) at the Anatomy Laboratory Toward Visuospatial Surgical Innovations in Otolaryngology and Neurosurgery (ALT‐VISION) at the Wexner Medical Center of The Ohio State University. All authors were certified by local regulatory agencies dealing with the use of human tissues and cadaveric studies. Each specimen underwent a high‐resolution CT scan and its digital data were imported to a surgical navigation system (Stryker Corporation; Kalamazoo, Michigan).

A 0^0^ endoscope (4‐mm diameter, 18‐cm length) coupled to a high definition camera and monitor (Karl Storz Endoscopy, Tuttlingen, Germany) was utilized to provide visualization throughout the dissections. All dissections were documented with video recordings and images that were archived for analysis (AIDA system Karl Storz Endoscopy, Tuttlingen, Germany). Still photographs documenting the anatomic relationships were correlated with multiplanar CT views provided by the image guidance system. A high‐speed drill (Stryker Co., Kalamazoo, Michigan) with straight hand‐piece and 3 to 4 mm coarse diamond burrs was used for bone removal and dissection.

## 3 RESULTS

**Figure 1:**
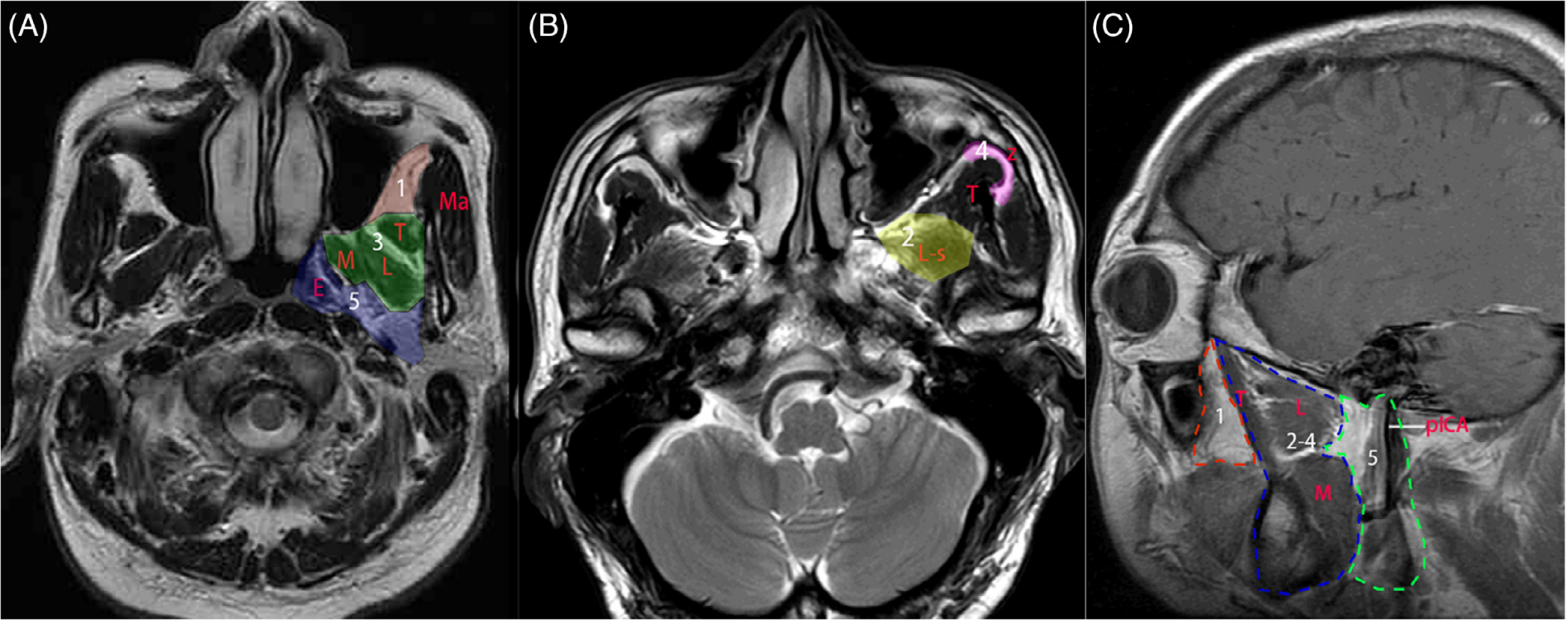
Schematic demonstrating of zones 1 to 5 on MRI scans. A, Zone 1, zone 3, zone 5; B, Zone 2, zone 4; C, Zone 1 (red dotted lines), zone 2 to zone 4 (blue dotted lines), and zone 5 (green dot lines) on sagittal image. M, medial pterygoid muscle; L, lateral pterygoid muscle (inferior head); T, temporalis muscle; Ma, masseter muscle; z, zygomatic arch; E, Eustachian tube; L‐s, superior head of lateral pterygoid muscle; pICA, parapharyngeal internal carotid artery [Color figure can be viewed at wileyonlinelibrary.com]

Following an endoscopic medial maxillectomy and Denker's approach, a progressive dissection of the ITF, including the masticator and the upper parapharyngeal spaces, was completed. Both techniques have been described in detail in previous studies.^20^, ^21^ A posterior nasal septectomy was performed to facilitate a bi‐nostril, four‐handed technique. According to potential anatomical spaces, we subdivided the ITF into five different zones. A schematic description was illustrated in Figure 1.

**Figure 2:**
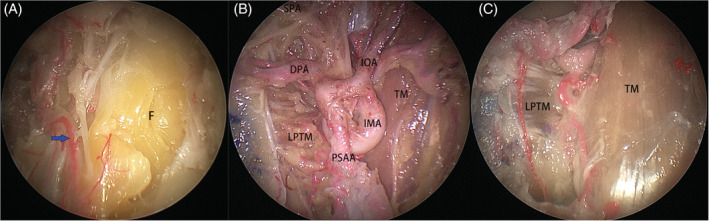
A, After removal of the posterolateral wall of the maxillary sinus and its periosteum, fat (F), and branches of the posterosuperior alveolar artery (PSAA, blue arrow) are identified; B, The main branches of the internal maxillary artery (IMA); C, Part of the buccal fat pad was removed to expose the temporalis muscle (TM) and lateral pterygoid muscle (LPTM). IOA, inferior orbital artery; DPA, descending palatine artery [Color figure can be viewed at wileyonlinelibrary.com]

**Figure 3:**
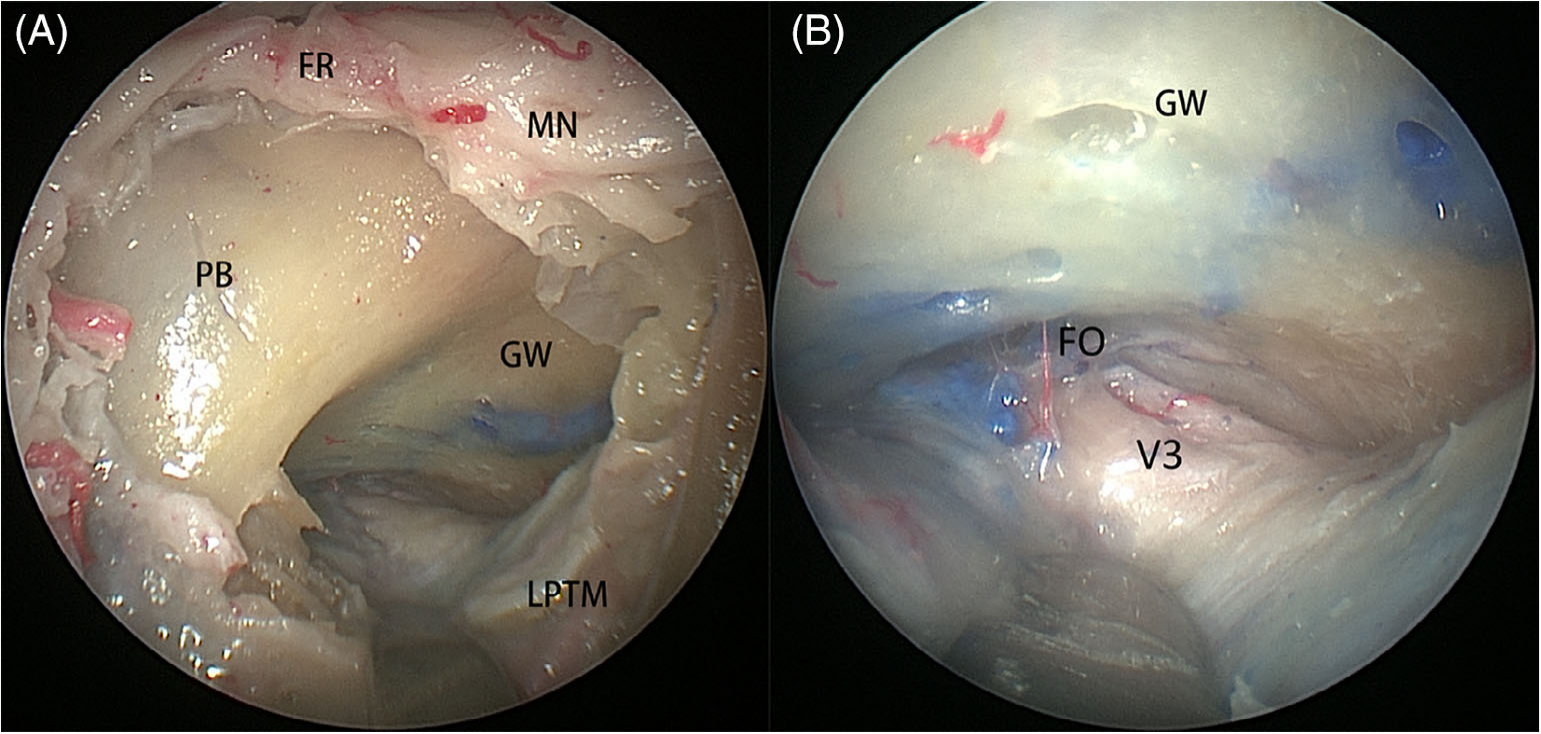
A, The maxillary nerve (MN) could be followed to identify the foramen rotundum (FR), pterygoid base (PB), and greater wing of sphenoid bone (GW), the superior head of lateral pterygoid muscle (LPTM) was elevated from GW in a subperiosteal plane; B, The foramen ovale (FO) and V_3_ are visualized [Color figure can be viewed at wileyonlinelibrary.com]

**Figure 4:**
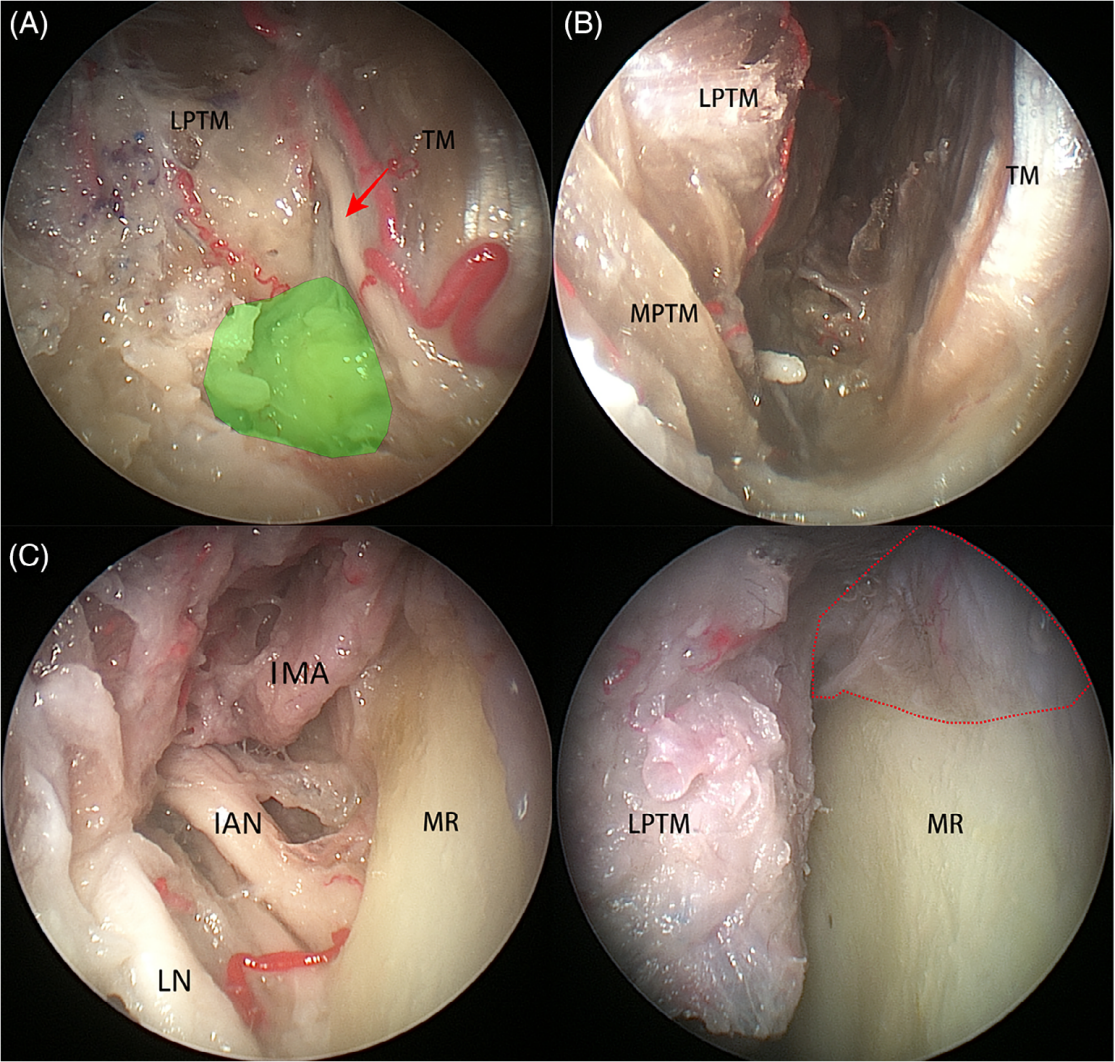
A, The deep temporal nerve (arrow) is consistently located at the medial border of temporalis muscle (TM), which is the landmark for identification of entrance of zone 3 (highlighted portion); B, Zone 3 was enclosed by the medial (MPTM) and lateral pterygoid muscles (LPTM) and TM; C, the lingual nerve (LN), inferior alveolar nerve (IAN), internal maxillary artery (IMA) and mandible ramus (MR) could be detected through this space; D, the medial aspect of the MR and fascia of the deep head of masseter muscle (enclosed dot lines) may be accessed [Color figure can be viewed at wileyonlinelibrary.com]

**Figure 5:**
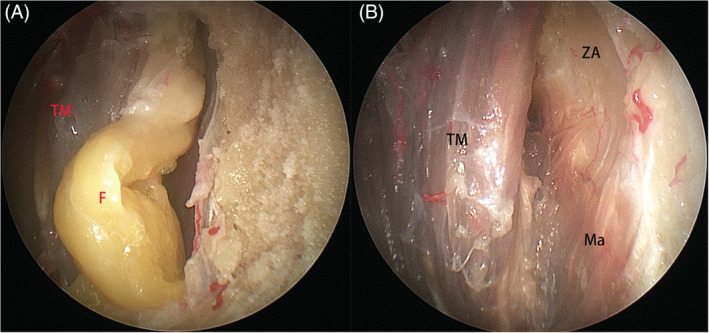
After removal of fat (F) in zone 4, A, the zygomatic arch (ZA) and the superficial head of the masseter muscle (Ma) could be exposed, B [Color figure can be viewed at wileyonlinelibrary.com]

**Figure 6:**
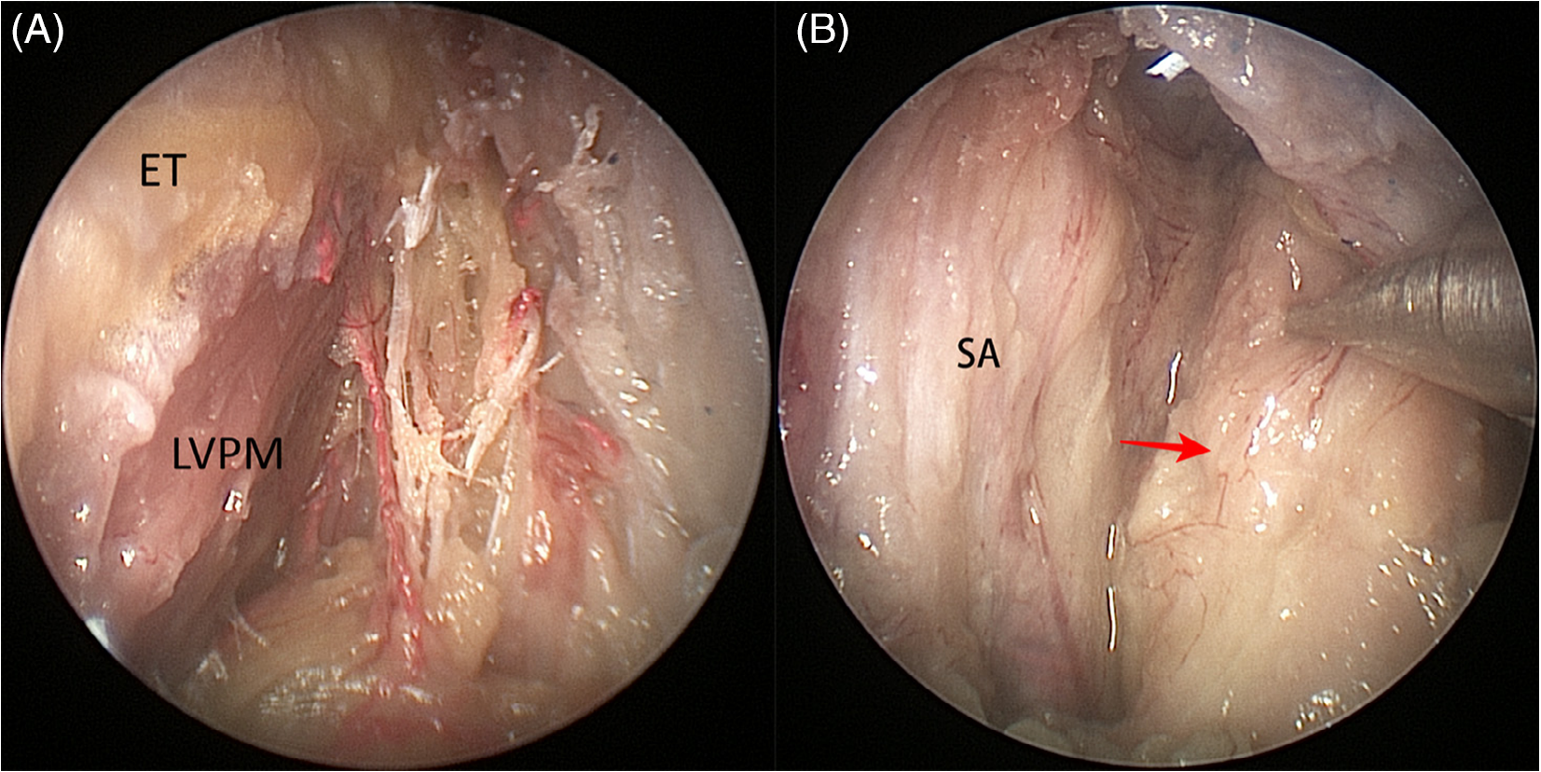
A, After transection of tensor veli palatini muscle, the Eustachian tube (ET) and levator veli palatini muscle (LVPM) could be identified; B, After removal of fat in pre‐styloid space, the deep lobe of parotid gland (arrow) could be viewed. SA, styloid aponeurosis [Color figure can be viewed at wileyonlinelibrary.com]

**Figure 7:**
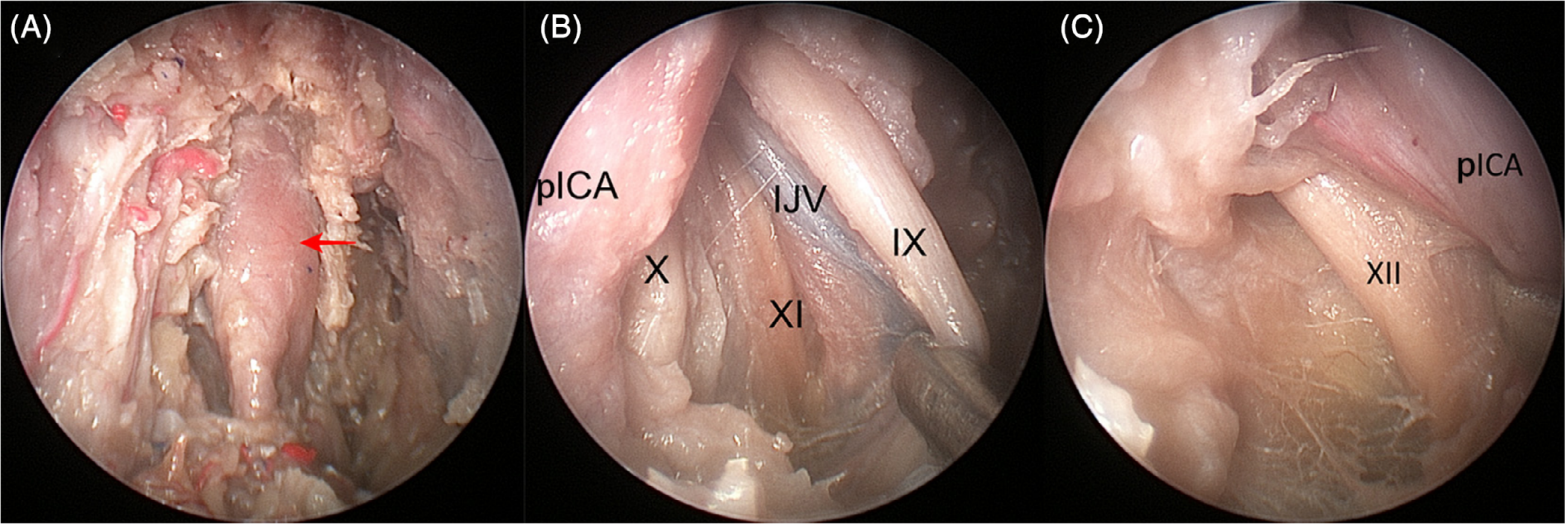
A, After removing the styloid aponeurosis (SA), the pICA could be visualized (arrow); B, Cranial nerves IX and X could be located between the pICA and internal jugular vein (IJV) and the cranial nerve XI is located posterior to the IJV; C, Cranial nerve XII is visualized posterior to the pICA [Color figure can be viewed at wileyonlinelibrary.com]

- *Zone 1* (*retromaxillary space*) was defined as the space lying between the posterolateral wall of maxillary sinus and the complex of temporalis and pterygoid muscles. After identification of the infraorbital nerve at the orbital floor, the posterolateral wall of the maxillary sinus medial to which was removed to expose the structures in pterygopalatine fossa. The remaining posterolateral wall of the maxillary sinus and its periosteum lateral to the infraorbital nerve was subsequently removed downward flush to the level of the floor of the maxillary sinus to expose the buccal fat pad (Figure 2A), which was carefully taken off to expose the branches of the internal maxillary artery (Figure 2B). The vascular branches were subsequently sacrificed to expose the temporalis and pterygoid muscles (Figure 2C).
- *Zone 2* (*superior interpterygoid space*) is located at the superior part of the ITF and comprises the superior head of the lateral pterygoid muscle, V_3_, and foramen ovale (Figure 3A). After careful dissection in the pterygopalatine fossa, the maxillary nerve, pterygopalatine ganglion, and greater palatine nerve were well preserved in all 12 sides. Using the maxillary nerve as a landmark to identify the pterygoid base and greater wing of the sphenoid bone, the superior head of the lateral pterygoid muscle was elevated from the greater wing of the sphenoid following a sub‐periosteal plane (Figure 3A). V_3_ and foramen ovale were identified posterior to the origin of the lateral pterygoid plate (Figure 3B).
- *Zone 3* (*inferior interpterygoid space*) includes the inferior head of the lateral pterygoid muscle, the medial pterygoid, and temporalis muscles. The deep temporal nerve, located at the medial border of the temporalis muscle (Figure 4A) in all 12 sides, serves as a landmark to identify *Zone 3* (Figure 4B). After displacing the temporalis muscle laterally, along the space enclosed by the temporalis muscle and the medial and lateral pterygoid muscles in a posterolateral direction, the lingual and inferior alveolar nerves lie on the superior border of the medial pterygoid muscle, and the internal maxillary artery is detected to enter the posterior aspect of the ITF (Figure 4C). In addition, most of the medial aspect of mandible ramus and the fascia of the deep head of masseter muscle could be exposed under guidance of 0^0^ scope through this corridor (Figure 4D).
- *Zone 4* (*temporo‐masseteric space*) is defined as the space lateral to the temporalis muscle, and mainly contains fat (Figure 5A). The medial aspect of the zygomatic arch and the superficial head of masseter muscle could be reached through this space (Figure 5B).
- *Zone 5* (*tubopharyngeal space*) includes the Eustachian tube, the tensor and levator veli palatini muscles and the structures within the UPPS. The neurovascular structures such as the pterygopalatine ganglion, vidian nerve, greater and lesser palatine nerves, descending palatine, and sphenopalatine arteries were sacrificed to enhance exposure. After elevation of the lateral pterygoid muscle off the lateral pterygoid plate, drilling of the pterygoid process, and lateral pterygoid plate was performed. Along the superior border of the medial pterygoid muscle, in a posterior direction, the tensor veli palatini muscle at the anterolateral aspect of cartilaginous Eustachian tube was identified and resected, and the levator veli palatini muscle at its anteroinferior aspect was exposed (Figure 6A). The fat in the pre‐styloid compartment was removed to expose the deep lobe of the parotid gland (Figure 6B). Removal of the styloid aponeurosis exposed the parapharyngeal internal carotid artery (pICA) (Figure 7A). In addition, the inferior cranial nerves (IX to XI) and the internal jugular vein may be visualized (Figure 7B). The hypoglossal nerve (XII), however, is posterior to the pICA; thus, its visualization requires mobilizing the vessel (Figure 7C).

## 4 DISCUSSION

Despite significant advances in optics, instrumentation and endonasal techniques, the management of lesions in the ITF via an endonasal endoscopic approach still presents a surgical challenge.^15^ Surgical approaches to the ITF based on pre‐existent pathways represented by potential aforementioned anatomical spaces help to spare unaffected tissues.^15^, ^22^ Furthermore, maximal preservation of normal adjacent structures (“innocent bystanders”) when a lesion is restricted to one specific region maintain postoperative function and quality of life. In the present study, dissection of *zones 1‐4* did not require any manipulation of neurovascular structures within the pterygopalatine fossa; however, dissection of *zone 5*, required its mobilization or sacrifice as it involves drilling of the pterygoid process and elevation of the inferior head of the lateral pterygoid muscle off the lateral pterygoid plate.

The aforementioned classification of ITF corridors, based on the muscles of mastication and their innervation, provides clinically relevant guidelines to access pertinent areas. *Zone 1* (*retromaxillary space*) contains fat and branches of the internal maxillary artery (pterygoid muscles are located at posterior aspect) and is best accessed by an endoscopic endonasal approach (prelacrimal or transantral). A case series by Zhou et al included the resection of a tumor that was exposed after the resection of the posterolateral wall of maxillary sinus, in accordance to the description of *zone 1* suggested in this study.^23^ Moreover, *zone 1* also constitutes a surgical corridor for management of lesions extending from nasal cavity into the anterior aspect of the ITF via pterygopalatine fossa (eg, angiofibroma).^24^

*Zone 2*, or *superior interpterygoid space*, correlates intimately with foramen ovale, V_3_, and the superior head of lateral pterygoid muscle. Theoretically, this zone should be a relatively common site of origin for neural tumors such as schwannoma.^4^ In addition to the management of lesions that originate from V_3_, endoscopic access into *Zone 2* also incorporates a corridor that can reach the middle cranial fossa and Meckel' cave (through the anterolateral triangle enclosed by V_2_ and V_3_).^25^

*Zone 3*, *or inferior interpterygoid space*, corresponds to the potential space enclosed by the inferior head of lateral pterygoid, medial pterygoid and temporalis muscles containing fat, and branches from the internal maxillary artery and V_3_. The lingual nerve and inferior alveolar nerve, arising from the posterior trunk of V_3_ travel through this space, and also may spring neural related tumors.^9^ Moreover, this space communicates with the medial aspect of mandible ramus and fascia of the deep head of masseter muscle; therefore, *Zone 3* provides a natural corridor to access lesions originating or extending into the medial aspect of the mandible ramus obviating the need for sacrificing any of the masticator muscles (ie, inferior head of lateral pterygoid muscle, medial pterygoid, or temporalis muscles).^26^ In cadaveric dissection, the removal of posterolateral wall of the maxillary sinus downward flush to level of the maxillary floor will enhance the exposure of *Zone 3*. For lesions arising in the lower part of the ITF around the angle of mandible, however, we identified that the floor of the nasal cavity will limit the exposure and the usage of straight instruments through the transnasal approach. For this kind of variation, the external transcervical or transparotid approach or the endoscopic transoral corridor with the assistance of robotics maybe an alternative selection.^14^, ^17^

*Zone 4*, or *temporo‐masseteric space*, lays between the temporalis muscle and zygomatic arch and mainly contains fat. One could access the medial aspect of the zygomatic arch and the superficial head of masseter muscle; however, lesions in this region are rare. This corridor, however, is often used as an avenue for the transposition of a temporoparietal fascia flap for the reconstruction in skull base.^27^ Under endoscopic guidance, the transposition of temporoparietal fascia flap via the corridor represented by *zone 4* has the potential of obviating the additional damage to structures within the ITF.

Due to its deep location, reaching *zone 5* (*tubopharyngeal space*) involves a complex surgical technique that requires the sacrifice of some normal structures such as the vidian nerve, pterygopalatine ganglion, greater and lesser palatine nerves, and terminal branches of the internal maxillary artery.^1^ In our cadaveric dissection, drilling of the pterygoid process and detachment of the lateral pterygoid muscle was necessary, the maxillary nerve and the medial pterygoid muscle could be preserved. Lesions in the post‐styloid compartment, including the pICA, cranial nerves IX to XII and internal jugular vein, are exposed only after removing the styloid aponeurosis.^28^, ^29^ Conversely, lesions originating in the pre‐styloid compartment do not require removing the styloid aponeurosis. Copious oozing is anticipated in this area; therefore, an endoscopic Denker's approach^21^ or an anterior maxillotomy or a variant is included to facilitate a four‐hands technique. The most common indication to approach *zone 5* includes benign lesions (ie, parapharyngeal space tumors) that originate within the space. Infiltration of the parapharyngeal space by a malignancy (eg, nasopharyngeal carcinoma), especially those that encroach the pICA, may be a contraindication for endoscopic endonasal corridor.^30^

To the authors' knowledge, this systematic approach to anatomical corridors to the ITF has not been reported before. However, we recognize that this study comprises significant limitations. It is a pre‐clinical work on normal cadaveric specimens, which cannot emulate the variety of anatomical changes brought by a tumor; thus, its utility still needs to be validated in live surgeries. Moreover, application of virtual reality techniques in future studies may help surgeons learn the anatomy in this complex area better. Furthermore, the classification of ITF is mainly indicated for endoscopic transnasal approaches; for transcervical or transoral approach to access the ITF, however, the utility of the classification system still deserves validation. In addition, the poorly pneumatized maxillary sinus will also limit the exposure of ITF via a transnasal approach.

## 5 CONCLUSION

The ITF can be divided into five zones based on spaces enclosed by the muscles of mastication. This paradigm provides guidance for the planning of endoscopic approaches to the ITF and helps to avoid damage to neurovascular structures and pterygoid muscles.

## CONFLICT OF INTEREST

The authors have no funding, financial relationships, or conflicts of interest to disclose.
